# Coconut ( *Cocos nucifera* L.) and Carob ( *Ceratonia siliqua* L.) Flours Dietary Fibers Differentially Impact Fecal Microbiota Composition and Metabolic Outputs In Vitro

**DOI:** 10.1002/fsn3.4724

**Published:** 2025-01-25

**Authors:** Seda Arioglu‐Tuncil, Dane Deemer, Stephen R. Lindemann, Yunus E. Tunçil

**Affiliations:** ^1^ Nutrition and Dietetics Department, Nezahat Keleşoğlu Health Sciences Faculty Necmettin Erbakan University Konya Türkiye; ^2^ Whistler Center for Carbohydrate Research, Department of Food Science Purdue University West Lafayette Indiana USA; ^3^ Department of Nutrition Science and Department of Biological Science Purdue University West Lafayette Indiana USA; ^4^ Food Engineering Department, Engineering Faculty Necmettin Erbakan University Konya Türkiye; ^5^ Edical and Cosmetic Plants Application and Research Center Necmettin Erbakan University Konya Türkiye

**Keywords:** 16S rRNA, alternative flours, colonic microbiota, fecal fermentation, gluten free flour, short‐chain fatty acids

## Abstract

Alternative flours can reveal beneficial health effects. The aim of this study was to evaluate and compare the effects of dietary fibers (DFs) of coconut and carob flours on colonic microbiota compositions and function. Coconut flour DFs were found to be dominated by mannose‐containing polysaccharides by gas chromatography (GC)/MS and spectrophotometer, whereas glucose and uronic acid were the main monosaccharide moieties in carob flour DFs. In vitro fecal fermentation analysis revealed that coconut flour DFs result in the generation of microbial butyrate as much as inulin does, which is known to be a butyrogenic prebiotic, but at a slower rate. Supportingly, coconut flour DFs promoted butyrate‐producing bacteria including *Roseburia* and *Coprococcus*, whereas carob flour DFs stimulated *Prevotella*‐related OTUs. In addition, higher microbial diversity was achieved at the end of the fermentation of coconut flour DFs by the fecal microbiota. This study clearly shows that alternative flours have distinct functionalities in terms of colonic microbiota composition and function, and coconut flour could be used as an alternative flour for the development of functional food products targeting colonic health.

## Graphical abstract

Effects of coconut and carob flour dietary fibers (DFs) on colonic microbiota composition and function were comparatively assessed in vitro. Coconut flour DFs result in generation of microbial butyrate as much as inulin does, which is known to be a butyrogenic prebiotic, but at a slower rate. Coconut flour DFs promoted butyrate‐producing bacteria including *Roseburia* and *Coprococcus*, whereas carob flour DFs stimulated *Prevotella*‐related OTUs.

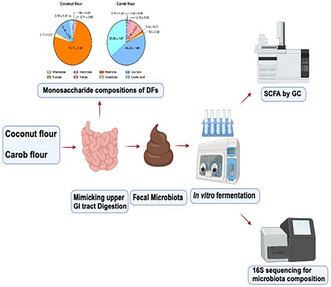

## 1 Introduction

Flour is the main ingredient for bakery products. Although cereal (wheat and corn) flours dominate the market, flours from alternative sources (i.e., legumes and fruits) have recently gained great attentions of consumers and producers due to their relatively higher nutritional value. Coconut flour and carob flour are two important alternative flours that have been widely used in the industry, especially for the productions of functional bakery products (Biernacka et al. 2017; Román et al. 2017; Sȩczyk et al. 2016; Vujić, Čepo, and Šebečić 2014).

Carob ( *Ceratonia siliqua* L.) flour is produced by drying, roasting, and grinding the carob pods from which the seeds are removed (Yousif and Alghzawi 2000). Carob pod is high in sugar (50%–65%), and the main sugars are reported to be sucrose, glucose, and fructose (Avallone et al. 1997). Due to its sweetness property, carob flour is widely used for bakery, confectionary, and beverage applications as well as being as cacao and chocolate substitutes (Srour et al. 2016). Its utilization as a cocoa substitute has continuously been increasing as it does not contain caffeine, theobromine, and oxalic acid. Unlike its sugar level, protein and lipid contents of carob flour were reported to be low, 3%–4%, and 0.4%–0.8%, respectively (Avallone et al. 1997). Carob flour contains 88.88% carbohydrates, 39.80% of which are dietary fibers (DFs) (Román et al. 2017). This high DF content of carob flour makes it suitable to be utilized in low glycemic index formulations. In addition, carob flour contains high amounts of phenolic compounds including flavanols, flavanones, and phenolic acids (Ortega et al. 2009; Papagiannopoulos et al. 2004; Stavrou, Christou, and Kapnissi‐Christodoulou 2018). Thus, carob flour is known to have a high antioxidant capacity. Furthermore, carob flour is a good alternative to wheat flour for celiac patients since it does not contain gluten.

Another type of alternative flour that does not contain gluten and have high DF content is coconut ( *Cocos nucifera* L.) flour. Coconut flour is the most widely used coconut product worldwide. It is produced by grating the coconut meat, which is followed by deoiling and milling. The white by‐product obtained after milling is called as coconut flour. Coconut flour was previously considered by‐products. Therefore, it used to be either utilized as an animal feed or discarded after coconut oil was extracted. With the discovery of rich nutritional content of coconut flour, its use in food applications as a functional ingredient has become popular. Coconut flour contains 3%–5% water, 4%–6% mineral matter, 8%–17% fat, 15%–22% protein, 56%–72% total carbohydrates, and 10%–56% DF (Raczyk, Kruszewski, and Michałowska 2021). It is also rich in micronutrients such as minerals (potassium, magnesium, selenium, iron) and essential amino acids (Raczyk, Kruszewski, and Michałowska 2021). Glycemic index (35) of coconut flour was reported to be lower than that of wheat flour (85) (Raczyk, Kruszewski, and Michałowska 2021). These nutritional properties of coconut flour make it a suitable substitute to wheat and corn flours in alternative functional food formulations.

Consumers' demands for gluten free and high DF containing diet has been increasing due to health‐related concerns. Thus, it is a common practice to mix different proportions of coconut flour or carob flour with wheat flour to increase the nutritional value (mainly DF contents) of the final product. The studies that came out from this perspective mainly focused on investigating the nutritional and rheological properties of the final products such as cookies, cakes, biscuits, and snacks (Abimbola n.d.; Hossain 2016; Ramya and Anitha 2020; Román et al. 2017; Sivakami and Sarojini 2013; Šoronja‐Simović et al. 2016; Sykut‐Domańska et al. 2020; Vujić, Čepo, and Šebečić 2014). However, studies focused on functionalities of their DFs are scarce.

DFs are defined as dietary substances (indigestible carbohydrates and lignin) that are resistant to digestion in the upper gastrointestinal tract of mammalians, and thus reach the colon, where they are partially or fully fermented by the residing microorganisms. Microorganisms found in the colon, called colonic microbiota, use DFs as carbon sources in anaerobic fermentation pathways, and as a result, they generate physiologically important metabolites, including, but not limited to, acetate, propionate, and butyrate. It is now well‐known that DFs have the ability to modulate colonic microbiota composition and metabolic output in a structure‐dependent way (Hamaker and Tuncil 2014). However, the impacts of DFs of coconut and carob flour on colonic microbiota compositions and microbial metabolites have not been elucidated yet. This study aimed to investigate and compare the gut microbiome modulation capabilities of DFs of coconut and carob flours through a series of in vitro fecal fermentation studies.

## 2 Materials and Methods

### 2.1 Proximate Analysis

Coconut flour and carob flour (Güzel Ada Gıda Mersin, Türkiye) were purchased from a local market. Samples purchased were stored at −20°C until further use. The moisture contents were determined by drying the coconut and carob flours to constant weights in a convection oven (Ecocell/EC 111, Germany) at 105°C ± 2°C overnight. The nitrogen contents of the seeds were determined according to the Dumas method using an automatic Nitrogen Analyzer (Leco Nitrogen Analyzer # FP828, St. Joseph, MI, USA). The protein concentrations were calculated by multiplying the nitrogen content with the conversion factor of 6.25. The total fat contents were determined by extracting the crude oils with hexane using an automated solvent extractor system (SER 148; Velp Scientifica, Usmate, Italy) at 130°C for 150 min. Ash contents were determined by weight difference after combustion of the flours at 550°C in a furnace (WiseTherm Digital Furnace, Wisd Laboratory Instruments) for at least 24 h. All proximate analyses were triplicated.

### 2.2 Simple Sugar Contents of the Flours

10 mg of flour was suspended in 1 mL of purified water, and vortexed for 5 min. The suspension was then filtered through a 0.45‐μm filter. 20 μL of filtrate was injected into the HPLC (Shimadzu‐ LC‐2050) coupled with a refractive index detector (RID) and a CarboSep column (Concise CHO87C), where H_2_O was used as a mobile phase with a flow rate of 0.4 mL/min and the column temperature was set to 85°C. Sucrose, glucose, and fructose at different concentrations were used to constitute the standard curves for quantification. The total simple sugar contents of the flours were calculated by summing up the weight percent of sucrose, glucose, and fructose.

### 2.3 Simulation of Upper Gastrointestinal Tract Digestion of the Flours

Coconut flour and carob flour samples were subjected to pepsin and pancreatin digestion, as previously described by Mishra and Monro (2009), to remove the digestible portions. The flow diagram of the upper gastrointestinal tract digestion procedure was previously published elsewhere (method A) (Tuncil et al. 2018). Samples that were subjected to in vitro upper gastrointestinal digestion procedure were stored at −20°C, and they were used for the subsequent analyses.

### 2.4 Determining the Neutral and Acidic Monosaccharide Compositions of DFs of Coconut and Carob Flours

Neutral monosaccharide compositions of the samples were determined according to the alditol acetate method described by Pettolino et al. (2012) using gas chromatography (GC)/MS, and uronic acid contents were measured spectrophotometrically as described by the AACCI (Uppsala Method) with the following modifications. Briefly, samples (5 mg) were first hydrolyzed with 72% sulfuric acid (250 μL) for 1 h at room temperature, followed by addition of 2.75 mL of water and further hydrolyzation at 100°C for 3 h. 100 μL of undiluted ammonia were then added to neutralize the samples. For neutral monosaccharide composition determination, 100 μL of hydrolyzed sample was then transferred into a clean tube followed by drying under nitrogen, reduction, and acetylation as described by Pettolino et al. (2012). Alditol acetate derivates were then injected into GC/MS. GC/MS conditions used are given elsewhere (Şahin et al. 2023). For uronic acid quantification, 100 μL of the hydrolyzed sample was transferred into a clean tube, followed by additions of 100 μL of NaCl/boric acid solution, 1.6 mL of 18 M sulfuric acid, incubation at 70°C for 40 mins, and addition of 100 μL 3,5‐dimethylphenol (100 mg diluted to 100 mL with glacial acetic acid). The absorbances of the samples were determined at 400 nm and 450 nm 10–25 min after addition of 3,5‐dimethylphenol, as described by AACCI. Neutral monosaccharide and uronic acid determinations were duplicated for each flour sample.

### 2.5 In Vitro Fecal Fermentation

In vitro fecal fermentation was performed in an anaerobic chamber (BACTRON300 Anaerobic Chamber; Shel Lab, Cornelius, OR) supplied with 90% N_2_, 5% H_2_, and 5% CO_2_, as previously described by Tuncil et al. (2018). Briefly, 50 mg of flour samples that were subjected to upper gastrointestinal tract digestion and positive controls inulin from chicory (Sigma #I2255) were weighed into 25 mL Balch tubes (Chemglass Life Sciences, Vineland, NJ) for each time point (0, 6, 12, and 24). As negative control, tubes containing no substrate were prepared for each time point. The tubes were sterilized by autoclaving at 121°C for 20 min and transferred into the anaerobic chamber. 4 mL of sterilized and reduced carbonate‐phosphate buffer was added on each tube and left for overnight for hydration. The next day, fecal samples were collected from three healthy donors who were consuming their routine diets, had not taken antibiotics for at least 3 months, and had a normal body mass index (BMI; 18.5 < BMI < 25 kg/m^2^). The collected fecal samples were tightly sealed in fecal sample collection containers, kept on ice, and immediately transferred in anaerobic chamber and used within 2 h of collection. Collected fecal samples were homogenized with 3 volumes of carbonate‐phosphate buffer and filtered through four layers of cheese cloth. Equal volume of fecal slurry obtained were then pooled, and 1 mL of pooled fecal slurry was inoculated into each tube. The tubes were then immediately closed with butyl rubber stoppers (Chemglass Life Sciences # CLS‐4209‐14), hermetically sealed with aluminum seals (Chemglass Life Sciences #CLS‐4209‐12) and incubated at 37°C in a shaking incubator (150 rpm). All fermentations were performed in triplicate. Protocols involving human stool collection and use were approved by the Scientific Research Ethics Committee of Health Sciences of Necmettin Erbakan University (application # 14567; approval # 2023/463).

At the end of each time point, two aliquots were collected from each tube for short‐chain fatty acid (SCFA) analysis (0.8 mL) and DNA extraction (1.6 mL). Samples collected for SCFA analysis were immediately mixed with a 200 μL of internal standard mixture (prepared by combining 157.5 μL of 4‐methylvaleric acid, 1.47 mL of 85% phosphoric acid, and 39 mg of copper sulfate pentahydrate in a final volume of 25 mL of ultrapure water) and stored at −20°C until further use.

### 2.6 SCFA Analysis

Microbial SCFAs generated by fecal microbiota throughout the fermentation process were quantified using a GC connected to a flame ionization detector (FID) (Shimadzu GC2030) possessing a fused silica capillary column (Restek Stabilwax Column), as previously described (Tuncil et al. 2018).

### 2.7 DNA Extraction, 16S rRNA Sequencing, Sequence Processing, and Community Analysis

The samples collected at 0 and 24 h time points were thawed at room temperature and centrifuged at 13,000 rpm for 10 min at 4°C. The supernatant was discarded, and DNA extraction was carried out on the pellet obtained after centrifugation using chemoenzymatic lysis approaches described Ferrera et al. (2010) and modified by Lindemann et al. (2013) with bead‐beating phenol‐chloroform approaches described previously for fecal samples by Mackenzie et al. (2015). The DNA extraction method details were provided step by step in our previous report (Tuncil et al. 2018).

The V4–V5 region of the 16S rRNA gene was amplified using the universal primers 515‐FB and 926‐R, followed by barcoding using Tru‐Seq barcoding primers, as previously described in our previous publication (Tuncil et al. 2018). Barcoded samples were sequenced on an Illumina MiSeq run with 2 × 250 cycles and V2 chemistry (Illumina Inc., San Diego, CA, USA) at the Purdue University Genomic Core Facility (West Lafayette, IN, USA). Sequences were processed using Mothur version 1.48.0 according to the Mothur MiSeq SOP (http://mothur.org/wiki/miseq_sop/) (Kozich et al. 2013; Schloss et al. 2009).

### 2.8 Statistical Analysis

Statistical analysis and data visualizations were done on GraphPad Prism version 10 for Mac OS X (GraphPad Software Inc., La Jolla, CA, USA). Data are presented as the mean ± SE. Significant differences among the samples and controls were determined through analysis of variance (ANOVA) at *α* = 0.05 significance level. Tukey's multiple comparison test at the *α* = 0.05 level was applied to determine whether mean differences were statistically different. Statistical differences in β‐diversity indexes were determined using the analysis of molecular variance (AMOVA) test implemented in Mothur.

## 3 Results and Discussion

### 3.1 Coconut and Carob Flours Differ in Their Proximate Contents and DF Compositions

Coconut flour was found to contain significantly (*p* < 0.05) higher protein (14.80%) and oil (11.20%) contents, compared to carob flour (3.73% and 0.36%, respectively), whereas carob flour was found to possess significantly (*p* < 0.05) higher total simple sugar content (31.62%), compared to coconut flour (21.20%) (Table 1).

**TABLE 1:** Proximate compositions of coconut and carob flours.

Components	Content (%, wet weight)*	
	Coconut flour	Carob flour
Moisture	4.68 ± 0.31^b^	6.01 ± 0.68^a^
Protein	14.80 ± 0.00^a^	3.73 ± 0.00^b^
Oil	11.20 ± 0.07^a^	0.36 ± 0.00^b^
Ash	2.45 ± 0.03^a^	2.51 ± 0.06^a^
Total simple sugar**	21.20 ± 0.09^b^	31.62 ± 1.79^a^
Sucrose	20.88 ± 0.17^b^	25.08 ± 1.38^a^
Glucose	nd	3.71 ± 0.38
Fructose	0.32 ± 0.09^b^	2.83 ± 0.05^a^

*Note:* Mean values of each component were compared between the flours, and those with different letter are significantly different (*p* < 0.05, two‐tailed student's *t*‐test).

Abbreviation: nd, not detected.

* Data are presented as mean ± SD (*n* = 3).

** Total simple sugar was calculated by summing up sucrose, glucose, and fructose contents.

**FIGURE 1:**
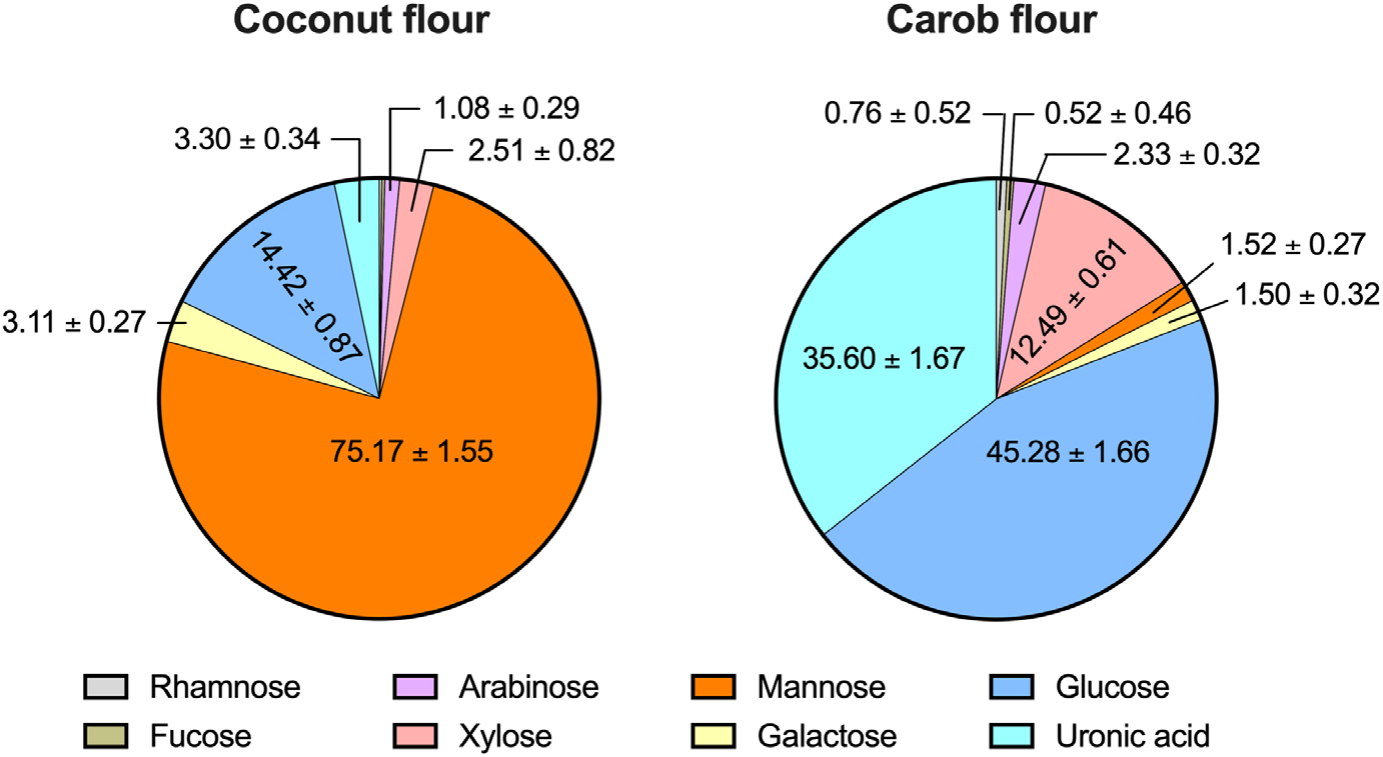
Monosaccharide compositions of DFs of coconut and carob flours (%, weight basis). Data are expressed as mean ± SD (*n* = 3).

The monosaccharide composition analyses revealed that coconut flour DFs are dominated by mannose residues (75.17%), followed by glucose (14.42%), uronic acid (3.30%), galactose (3.11%), xylose (2.51%), and arabinose (1.08%) (Figure 1), indicating that coconut flour DFs are composed mainly of mannans. This agrees with Du et al. (2021) who reported that mannose is the major (82.75%) monosaccharide moiety presented in coconut flour DFs. On the other hand, carob flour DFs were found to be composed mainly of glucose (45.28%), uronic acids (35.60%), and xylose (12.49%) (Figure 1). Due to the structural differences, DFs from coconut and carob flours are expected to have varied functional properties and reveal differential impacts on colonic microbiota composition and function. The monosaccharide composition of carob flour DFs reported here disagrees with Petkova, Ivanov, and Mihov (2017) who reported that the main monosaccharide moieties found in polysaccharides of commercially available carob flour are mannose (70.5%, mol basis), galactose (20.1%, mol basis), and xylose (10.5%, mol basis). This discrepancy between studies could be attributed to the fact that, although carob flours are generally produced from carob pod from which the seeds are removed, some carob flours might contain its seed fraction, which is rich in mannose‐based polysaccharides (mainly galactomannans) (Dakia 2011). Thus, the carob flour used in Petkova, Ivanov, and Mihov (2017)'s study might contain carob seed, while the carob flour used in this study might be produced from carob pods from which the seeds are removed. These further indicate that commercially available carob flours are not standardized and perhaps have different functional properties.

**FIGURE 2:**
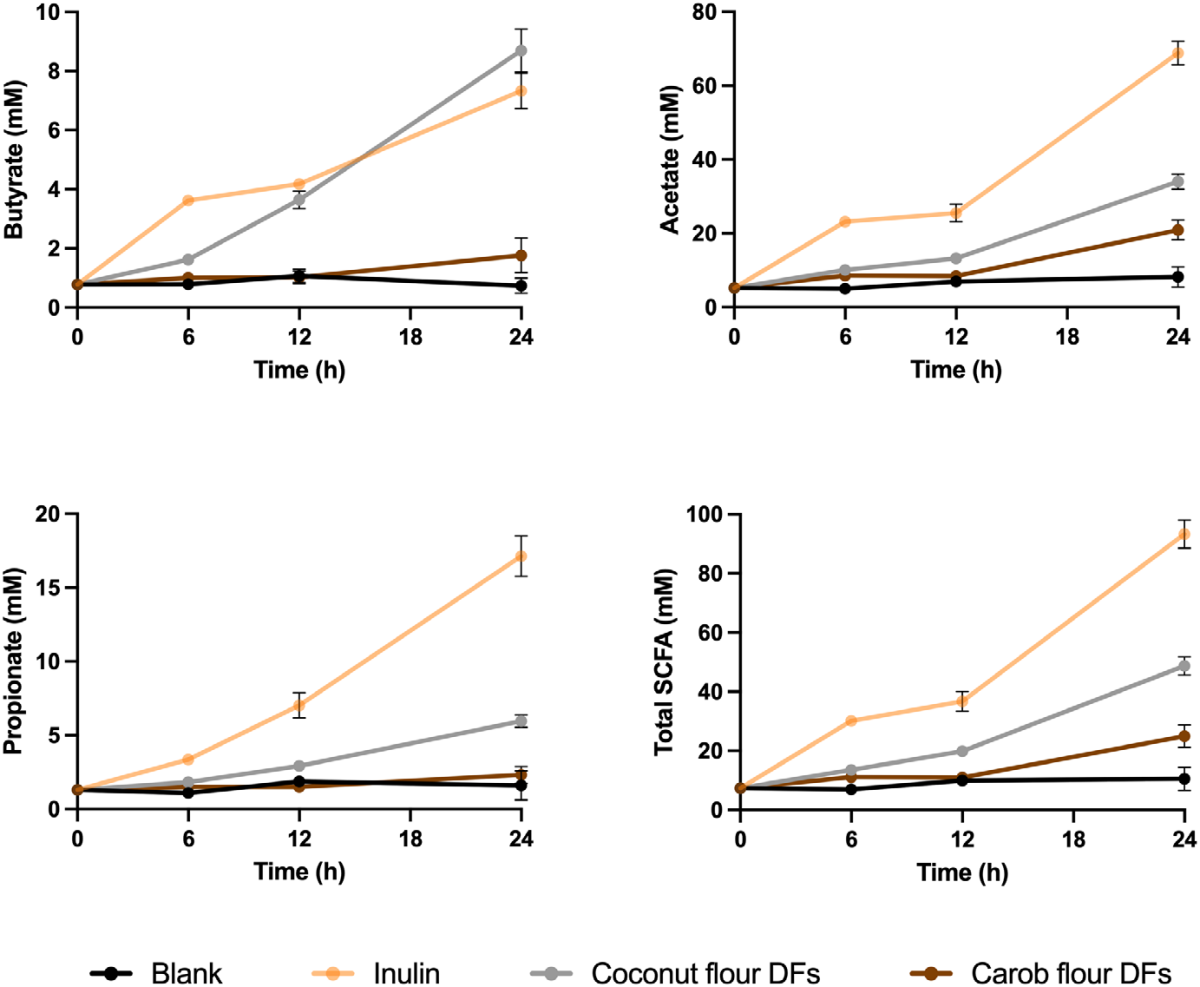
Short‐chain fatty acids (SCFAs) productions by fecal microbiota throughout in vitro fermentations of DFs of coconut and carob flours. Inulin was included as a fast fermentable, butyrate‐producing control. The blank did not contain any carbon source (negative control). Total SCFA amounts were calculated by summing up the acetate, propionate, and butyrate amounts. Error bars represent the standard deviation of the mean (*n* = 3).

### 3.2 Fermentation of Coconut Flour DFs Boosted Butyrate Generation by Fecal Microbiota

To investigate how coconut and carob DFs impact the microbial SCFAs in the colon, a series of in vitro fecal fermentation assays were performed, and butyrate, acetate, and propionate generated by fecal microbiota were monitored throughout the fermentation assay (Figure 2). As a positive control, inulin, which is recognized as a rapid fermentable butyrate‐boosting prebiotic, was utilized. Our results revealed that fermentation of coconut flour DFs resulted in a generation of similar amount of butyrate that was observed in inulin, indicating that coconut flour DFs is as butyrogenic as inulin. In addition, the acetate:propionate:butyrate ratio was 74:18:8 at the end of inulin fermentation, whereas that was 70:12:18 at the end of coconut flour DFs fermentation, further confirming the high butyrate‐boosting capacity of coconut flour DFs. This is also in agreement with the results of de Godoy et al. (2015) who reported that coconut endosperm fibers have great ability to promote butyrate generation in an in vitro fecal fermentation system. Butyrate has gained an increasing attention as it has many health‐promoting effects both locally and systematically for human body including being energy source of colonocytes, reducing inflammation, protecting from colon cancer, and maintaining of intestinal homeostasis. Interestingly, at early stage of the fermentation (in the first 6 h of fermentation), the butyrate level observed in inulin sample was significantly (*p* < 0.05) higher than that was observed in coconut flour DFs sample, suggesting that butyrate generation by colonic microbiota as results of coconut flour DFs fermentation occurs at a slower rate, compared to that of inulin. This further suggests that, compared to inulin, coconut flour DFs can deliver butyrate, a beneficial microbial metabolite, to more distal parts of the colon, where the majority of the colon‐related diseases (for instance, inflammation, and colon cancer) occur.

However, carob flour DFs did not significantly (*p* > 0.05) change the propionate and butyrate levels but increased the acetate levels at the end of the fermentation period (Figure 2). In addition, total SCFA generated at the end of the fermentation period by fecal microbiota as a result of carob flour DFs fermentation was significantly (*p* < 0.05) lower than those of coconut flour DFs and inulin, suggesting that fermentation of carob flour DFs by colonic microbiota is harder than those of coconut flour DFs and inulin. This agrees with Zhu et al. (2019) who reported that carob fiber has low fermentability. These results collectively show that coconut and carob flour DFs distinctly impact the functionality of fecal microbiota.

### 3.3 Higher Microbial Diversity Was Observed in Coconut Flour DFs

**FIGURE 3:**
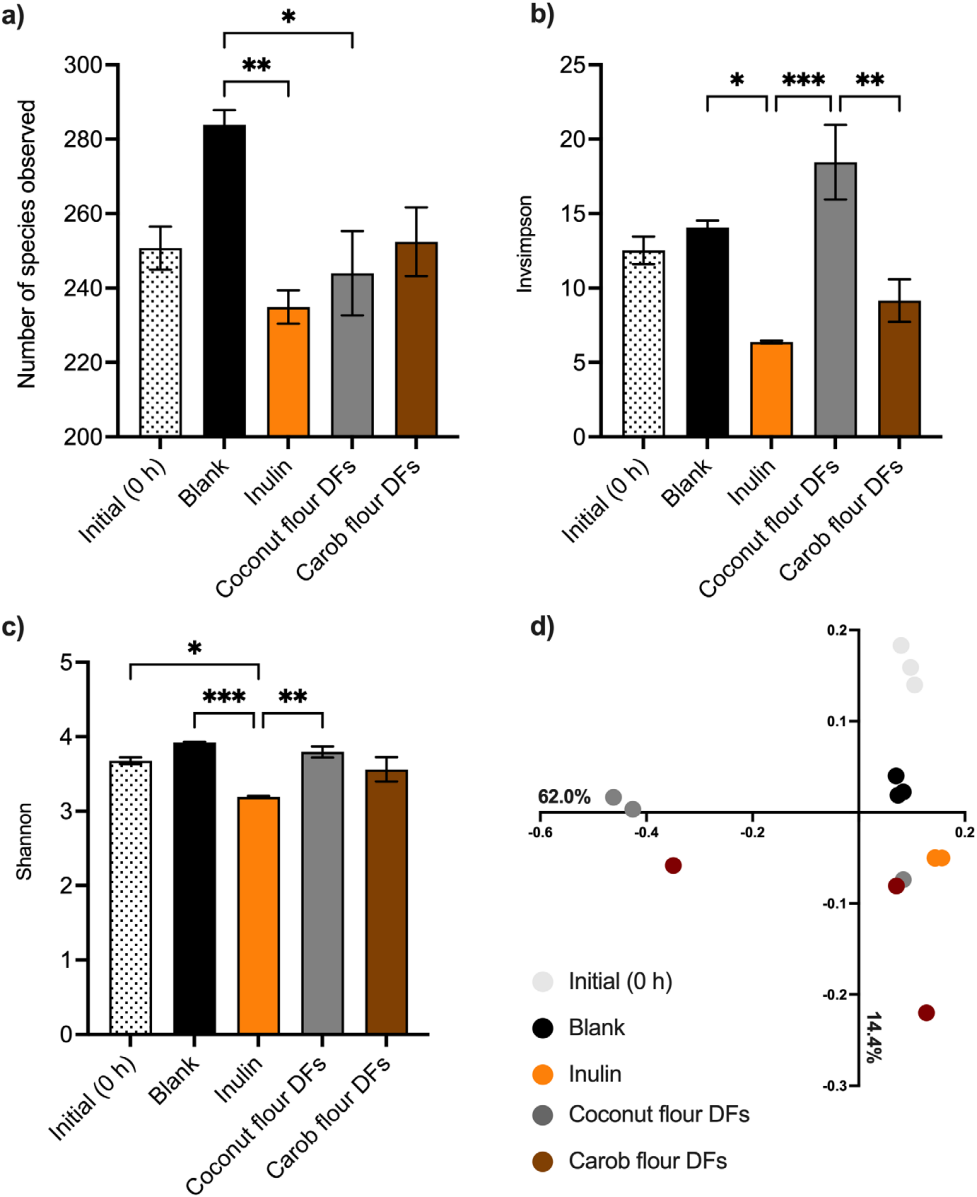
α‐Diversity (richness and/or evenness) of the microbial communities as measured by (a) the number of species observed (NSO), (b) inverse‐Simpson, and (c) Shannon indexes. Error bars represent the SE of the mean (*n* = 3). *p < 0.05, **p < 0.01, ***p < 0.001 (Tukey's multiple comparison test). (d) Principal coordinate analysis (PCoA) of Bray–Curtis dissimilarity of microbiota communities based on the relative abundances of OTUs at a 97% identity level. Inulin was included as a fast fermentable, butyrate‐producing control. The blank did not contain any carbon source (negative control).

α‐Diversity (richness and/or evenness) of the microbial communities was investigated by measuring the number of species observed (NSO), inverse‐Simpson, and Shannon indexes (Figure 3a–c). NSO accounts for species' richness, while inverse‐Simpson and Shannon indexes account for both species' richness and evenness. Community richness did not significantly (*p* > 0.05) vary between samples after the fermentation period. On the other hand, fermentation of coconut flour DFs resulted in significantly (*p* < 0.05) higher inverse‐Simpson index value than inulin and carob flour DFs. Similarly, at the end of the fermentation, significantly (*p* < 0.05) higher Shannon index value was observed in coconut flour DFs; however, the Shannon index value of coconut flour DFs did not significantly (*p* > 0.05) differ than carob flour DFs. The lower inverse‐Simpson value observed in inulin could be attributed to the relatively simple chemical structure of inulin composing of β‐(2, 1)‐linked fructose units that could possibly favor only limited number of bacteria, while coconut flour DFs and carob flour DFs compose of mixture of polysaccharides containing various monosaccharide moieties and linkages, thereby being carbon source for different (a higher number of) microbial species.

Overall structures of the fecal microbiota communities were compared using the Bray–Curtis dissimilarity test based on the relative abundances of OTUs at 97% similarity level. Although microbial communities of samples were overall clustered accordingly (Figure 3d), no significant differences were observed (AMOVA, *p* = 0.075). Relatively small sample set included in this study could be the reason why the differences were not statistically significant.

### 3.4 Coconut Flour DFs Promoted *Roseburia* and Coprococccus‐Related OTUs That Are Known to Be Butyrate Producers

**FIGURE 4:**
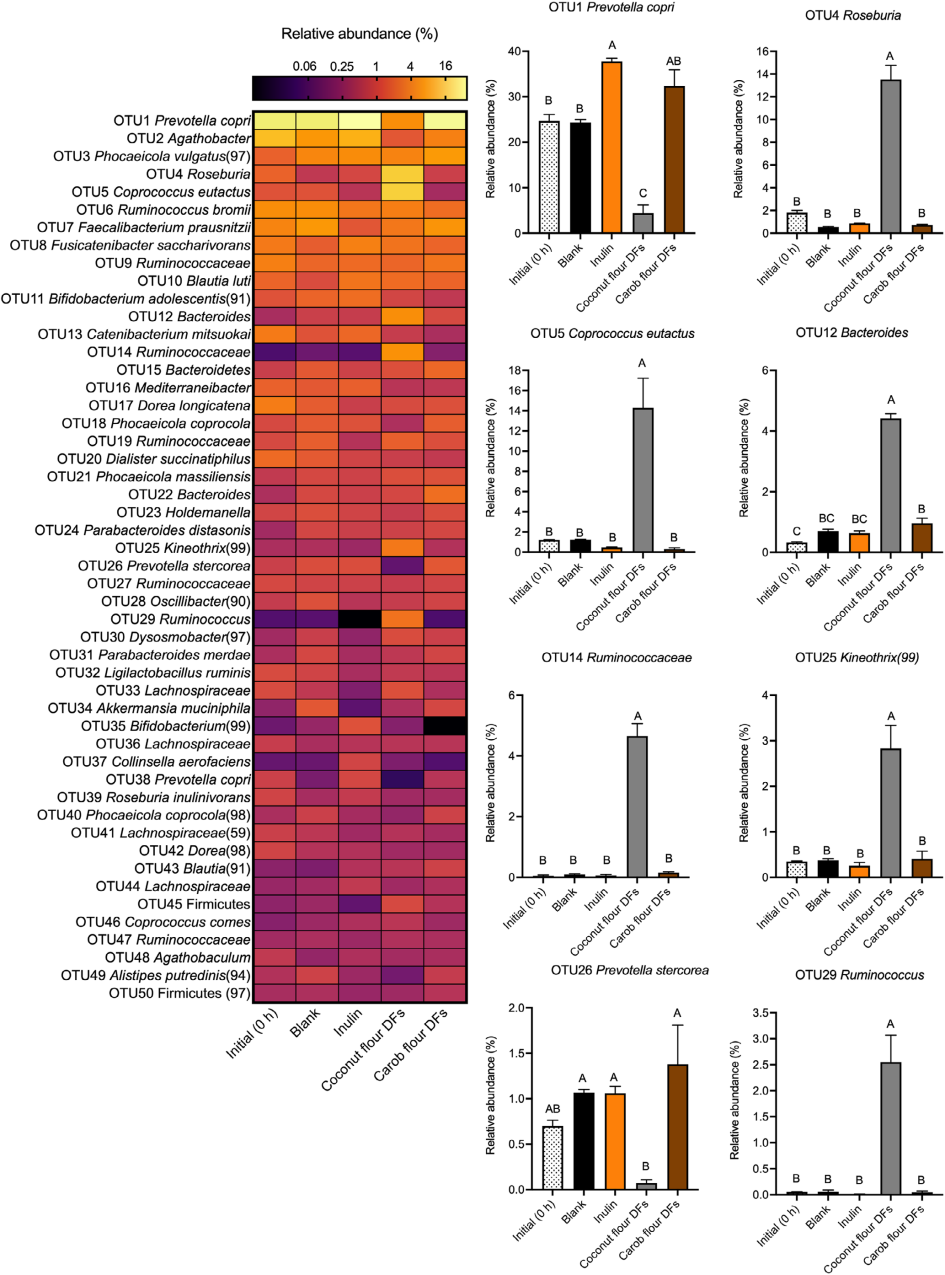
Relative abundances (percentage of sequencing) of OTUs before and after 24 h in vitro fecal fermentation. Heat map showing the relative abundances of top 50 OTUs before and after 24 h in vitro fermentation (left panel). Bar graphs showing the relative abundances of some OTUs before and after 24 h in vitro fermentation (right panel). Error bars represent the SE of the mean (*n* = 3). Mean values with different letter are significantly different (*p* < 0.05, Tukey's multiple comparison test). Inulin was included as a fast fermentable, butyrate‐producing control. The blank did not contain any carbon source (negative control).

The effects of coconut and carob flours DFs on fecal microbiota compositions were determined at the OTU level before and after (24 h post‐inoculation) fermentation (Figure 4). Our results revealed that fermentation of coconut flour DFs resulted in promotions of butyrate‐producing bacteria. Specifically, 7.5‐fold increases were observed in the relative abundance of OTU4 *Roseburia* at the end of the fermentation of coconut flour DFs (13.53%), compared to initial (1.81%). This is also in line with our SCFA data, where high butyrate formation by fecal microbiota was observed as a result of coconut flour DFs fermentation, as *Roseburia* spp. are among the butyrate‐producing bacteria found in the human colon. Abundance of *Roseburia* spp. was shown to be significantly lower in the presence of inflammatory diseases (Chassard et al. 2012; Takahashi et al. 2016) and colorectal cancer (Geng et al. 2013; Wang et al. 2012). In addition, abundance of *Roseburia* in the colon was reported to be inversely correlated with type 2 diabetes (Gurung et al. 2020), atherosclerosis (Karlsson et al. 2012), and atherogenesis (Kasahara et al. 2018). Similarly, fermentation of coconut flour DFs resulted in 12‐fold increases in the relative abundance of OTU5 *Coprococcus eutactus* (from 1.2% to 14.3%). *C. eutactus* is another butyrate‐producing bacteria found in the human colon that is proposed to reveal beneficial health effects; for example, *C. eutactus* has been shown to incite anti‐inflammatory cytokines such as IL‐4, IL‐5, and IL‐10 while suppressing the proinflammatory cytokines such as TNF‐α, IL‐1β, and IL‐6 and enhancing intestinal epithelial barrier function (Yang et al. 2022). Butyrate synthesis by *Roseburia* and *C. eutactus* occurs by conversion of acetate to butyrate by acetyl coenzyme A and butyryl coenzyme (Louis and Flint 2017). Thus, lower acetate and higher butyrate relative abundances observed at the end of coconut flour DFs fermentation (Figure 2) could be attributed to *Roseburia*'s and *C. eutactus* ' abilities to convert acetate to butyrate. The relative abundance of OTU25 *Kineothrix* (99) was also increased 8‐fold (from 0.35% to 2.83%) after coconut flour DFs fermentation. Genus *Kineothrix*, recognized among commensal gut bacteria, is characterized as a butyrate producer within the family *Lachnospiraceae* (Haas and Blanchard 2017).

In addition, coconut flour DFs treatment resulted in 51‐ and 93‐fold increases in the relative abundance of *Ruminococcus*‐related OTU (OTU29 *Ruminococcus*; from 0.05% to 2.55%) and the parent taxa (OTU14 *Ruminococcaceae*; from 0.05% to 4.66%), respectively. Although *Ruminococcus* spp. are not capable of producing butyrate, increase in their abundance is generally associated with higher butyrate levels (Sasaki et al. 2022; Teichmann and Cockburn 2021). This is mainly attributed to their great ability to produce acetate by metabolizing dietary substances that can be then converted to butyrate by butyrate‐producing bacteria (Baxter et al. 2019). Thus, high increases in the relative abundances of OTU14 *Ruminococcaceae* and OTU29 *Ruminococcus* could contribute to the high amount of butyrate production observed in this study.

A 6‐fold decreases were observed in the relative abundance of OTU1 *Prevotella copri* , as a result of fermentation of coconut flour DFs (from 24.7% to 4.4%). A decrease in its relative abundance as a result of coconut flour DFs fermentation could be attributed to increases in the relative abundance of other butyrate‐producing/boosting taxa. Conversely, carob flour DFs and inulin fermentation resulted in stimulation of *Prevotella*‐related OTUs (OTU1 and OTU26). *Prevotella* is one of the most abundant genera found in the human colon that has great ability to metabolize dietary carbohydrates. Genus *Prevotella* is characterized as a significant propionate and acetate producer via the succinate pathway (David et al. 2014; Oliphant and Allen‐Vercoe 2019; Wu et al. 2011). Therefore, a relatively higher amount of propionate generation were observed in inulin and carob flour DFs treatments could be attributed to their ability to stimulate *Prevotella* spp.

## 4 Conclusion

In this study, the effects of DFs of coconut and carob flours on colonic microbiota compositions and function were determined through an in vitro fecal fermentation assay. Fermentation of coconut flour DFs by fecal microbiota resulted in generation of a high amount of butyrate, whereas the same trend was not observed in carob flour DFs. More interestingly, coconut flour DFs were found to be as butyrogenic as inulin, which is known to be a butyrogenic prebiotic. Furthermore, butyrate formation in coconut flour DFs occurred at a slower rate than inulin, suggesting that, when consumed, compared to inulin, coconut flour DFs can deliver butyrate to the more distal region of the colon. Supportingly, coconut flour DFs promoted butyrate‐producing bacteria including *Roseburia* and *Coprococcus*, whereas carob flour DFs stimulated *Prevotella*‐related OTUs. These distinct effects of coconut flour and carob flour DFs on colonic microbiota composition and function were attributed to differences in their compositions, the former composed mainly of mannose‐based polysaccharides, while the latter contained glucose‐ and uronic acid‐based polysaccharides. This study clearly shows that not all alternative flours have the same functionality in terms of colonic microbiota, and coconut flour could be used as an alternative flour for the development of functional food products targeting colonic health. A limitation of this study is the relatively small sample size, which may limit the applicability of the results to a broader population. Future in vitro and in vivo studies with larger and more diverse groups of donors and populations should be performed to confirm these results. In addition, in this study, no attempt was made to test the dose‐dependent effects of coconut and carob flours DFs on colonic microbiota composition and function, which could be important for assessing their potential use in designing functional foods. Future studies should focus on addressing this gap.

## Author Contributions

**Seda Arioglu‐Tuncil:** conceptualization, formal analysis, funding acquisition, methodology, investigation, visualization, interpretation, project administration, supervision, writing – original draft, writing – review and editing. **Dane Deemer:** investigation, writing – review and editing. **Stephen R. Lindemann:** formal analysis, writing – review and editing. **Yunus E. Tunçil:** formal analysis, methodology, investigation, visualization, interpretation, writing – original draft, writing – review and editing.

## Conflicts of Interest

The authors declare no conflicts of interest.

## Data Availability

The data that support the findings of this study are available on request from the corresponding author.
